# Impairments to Thermoregulation in the Elderly During Heat Exposure Events

**DOI:** 10.1177/2333721420932432

**Published:** 2020-06-15

**Authors:** Alison Millyard, Joe D. Layden, David B. Pyne, Andrew M. Edwards, Saul R. Bloxham

**Affiliations:** 1 Plymouth Marjon University, UK; 2 University of Canberra, Australian Capital Territory, Australia; 3 Canterbury Christ Church University, UK

**Keywords:** ageing, perception, behavioral thermoregulation, heat acclimation, cooling strategies

## Abstract

Heat waves represent a public health risk to elderly people, and typically result in an increased rate of hospital admissions and deaths. Studies of thermoregulation in this cohort have generally focused on single elements such as sweating capacity. Sweating capacity and skin blood flow reduce with age, reducing ability to dissipate heat. Perception of effort during heat exposure is emerging as an area that needs further investigation as the elderly appear to lack the ability to adequately perceive increased physiological strain during heat exposure. The role of the gut and endotoxemia in heat stress has received attention in young adults, while the elderly population has been neglected. This shortcoming offers another potential avenue for identifying effective integrated health interventions to reduce heat illnesses. Increasing numbers of elderly individuals in populations worldwide are likely to increase the incidence of heat wave-induced deaths if adequate interventions are not developed, evaluated, and implemented. In this narrative-style review we identify and discuss health-related interventions for reducing the impact of heat illnesses in the elderly.

## Introduction

Tens of thousands of deaths have been caused by heat waves across Europe since 2000 (European Environment Agency [EFA], 2016). There are an estimated 1,500 heat-related deaths every year in the United States (Epstein et al., 2005). A health center in Paris recorded 2,814 deaths during the 2003 heat wave, 81% of these were in people aged >75 years (Epstein et al., 2005). Exposure to hotter than usual temperatures poses a thermoregulatory challenge to the human body, particularly when this occurs suddenly, precluding opportunities for acclimatization. Nevertheless, heat illness can be managed through simple behavior changes such as drinking more water and seeking shelter in air-conditioned buildings (Harduar Morano et al., 2016). Such behavioral strategies rely on effective efferent-afferent physiological responses, but these have been shown to decrease with aging. Weather-related heat deaths demonstrate that discrete groups of the population cannot cope effectively with extreme temperatures. The elderly in particular have been identified as a subpopulation at risk during extreme heat weather events (Åström et al., 2011).

In 2017, approximately 18% of the U.K. population was aged >64 years; this is expected to increase to around 24% by 2037 (Office of National Statistics, 2017). The number of people worldwide aged >64 years is predicted to increase from 703 million in 2019, to 1.5 billion by 2050, making up 16% of the world’s population (United Nations Department of Economic and Social Affairs Population Division, 2019).

The cost of treating heat illness in the United States between 2001 and 2010 was US$650 million (Schmeltz et al., 2016) with 73,180 heat-related illness hospitalizations. In the 2006 California heat wave, the health care costs were estimated at US$179 million, not including the economic cost of 655 premature deaths of US$5.2 billion (Knowlton et al., 2011).

It is widely accepted within the scientific community that the Earth is getting warmer, mean surface temperature increased 0.5°C between 1979 and 2010, and extreme weather events, including heat waves, are becoming more frequent (American Meteorological Society, 2012; Met Office, 2018). With the combination of increasing heat waves and elderly population growth, the number of heat-related deaths is likely to increase. Further research into physiological, perceptual, and behavioral responses of older age population groups is now needed. This narrative-style review examines relevant studies on the interaction between the cardiovascular and immune systems and behavioral responses to heat stress in an elderly population. Evaluation of these studies will inform practitioners working with this population, and researchers investigating the effects of interventions aimed at reducing the impact of heat stress at both an individual and community level.

## Aging and Thermoregulation

**Figure 1.**
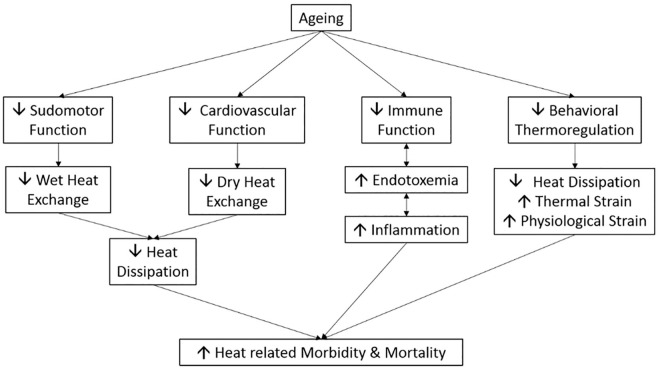
Factors contributing to increased risk of heat illness and death in aging.

Aging impacts thermoregulation in several ways (Figure 1), older adults (≥ 50 years) store 1.3 to 1.8 times more body heat when exposed to the same heat load than younger adults (19–30 years) during both exercising and passive heat exposure in both humid and dry conditions (35°C–44°C, 15%–30% relative humidity [RH]; Kenny et al., 2017; Larose et al., 2014; Stapleton et al., 2015a). The higher heat storage in the older individuals is due to a reduction in heat loss (Larose et al., 2014) caused by an attenuated sweat response (Kenny et al., 2017; Stapleton et al., 2015a) and increased dry heat gain (Kenny et al., 2017). These studies clearly show a reduced thermoregulatory function with aging.

### The Sudomotor System

Sweating is a critical mechanism for heat loss in humans, particularly when ambient temperature is above skin temperature as dry heat exchange results in heat gain in these situations. Sweating function declines with age at differing rates. Sudomotor function declines first in the legs, followed by progressive decrements in the upper body (Inoue et al., 2004). Reduced sweating capacity has been observed by age 40 years (Dufour & Candas, 2007; Larose et al., 2013). Loss of sweating capacity comes from reduced function of each sweat gland rather than a reduction in the number of sweat glands (Inoue et al., 2004), and is thought to be caused by local rather than central factors (Dufour & Candas, 2007). Older adults have a higher core temperature threshold for the onset of sweating, when exposed to 40°C and 40% RH, resting men aged >60 years started sweating at a core temperature of 37.0°C, whereas men aged <40 years started sweating at 36.7°C (Sagawa et al., 1988). This difference was also observed in females, with women aged 58 years sweating at core temperatures of 37.3°C and 37.5°C compared with 37.1°C and 37.2°C for women aged 23 years while exercising (3 × 30 min cycling at 250 W, 325 W, and 400 W) in the heat (40°C, 15% RH; Stapleton et al., 2015b).

The delayed onset of sweating coupled with the inability to increase and maintain a high sweat rate will delay the effect of cooling from sweat reducing its effectiveness, resulting in a higher core temperature and greater heat strain in the elderly. Individual sweating rates vary greatly. Sato and Sato (1983) reported sweat rates ranging from 0.8 to 10.1 nl/min per gland. Therefore, the decline in sweat response is likely to be highly individualized. Nitric Oxide (NO) stimulates sweat production in young adults (Amano et al., 2017; Stapleton et al., 2014). However, NO inhibition has little effect on sweat rate in men aged >60 years (McGarr et al., 2019; Stapleton et al., 2014). As high level of aerobic fitness increases sweating capacity in young and older adults (Fritzsche & Coyle, 2000; Tankersley et al., 1991), more work should focus on how aging and physical activity levels affect this relationship.

### The Cardiovascular System

With aging, the cardiovascular system experiences functional and structural changes (Edwards & Hettinga, 2018). Total blood volume decreases (Davy & Seals, 1994), reactive oxygen species increase and NO availability reduces, yielding a decrease in endothelial-dependent dilation and a reduced blood flow (Donato et al., 2015). These alterations put an added stress upon the cardiovascular system. The risk of death during a heat wave is increased in cardiovascular disease patients, with an odds ratio of 2.3–7.2 (Naughton et al. 2002; Semenza et al., 1996), and a relative risk of 2.0–2.4 (Kaiser et al., 2007; Wainwright et al., 1999). Dry heat transfer relies on the cardiovascular system redistributing blood toward the skin. Recent work has demonstrated that even in healthy active older adults free from cardiovascular disease, calf blood flow is attenuated during passive heat exposure (Kenny et al., 2017). Older adults increase their skin blood flow (SkBF) ~2–3 times less than their younger counterparts during passive (supine rest in water perfused suit at 50°C until thermal tolerance reached) and active (seated rest for 50 min, cycling for 20 min at 35% V̇O_2_max, 30 min at 60% V̇O_2_max in 36°C, 20% RH) heat exposure (Ho et al., 1997; Minson et al., 1998). This reduction in SkBF is due to a reduced stroke volume attenuating the increase in cardiac output (Minson et al., 1998). These cardiovascular differences were observed during passive heat exposure, without the additional demand of supplying exercising muscle with adequate blood flow. Attenuated SkBF will reduce dry heat loss, and therefore increase heat strain on the body. The elderly will struggle to dissipate heat effectively compared with their younger counterparts, resulting in increased thermal and physiological strain.

Inhibiting NO synthase in active older (61 years) men attenuates the increase in SkBF during passive and active heat exposure, and Ca^2+^ and adenosine triphosphate mediated K^+^ channels are dependent on NO synthase for increasing SkBF (McGarr et al., 2019). Increasing NO availability via nutritional intervention improves endothelial function in the elderly at rest in thermoneutral and whole-body heat stress conditions (de Oliveira et al., 2016; Stanhewicz et al., 2015). Increasing NO availability offers a potential mechanism to reduce heat stress by increasing SkBF and improving dry heat exchange. However, despite increasing absolute SkBF, increasing NO availability through folic acid supplementation (5 mg/day × 6 weeks) had no effect on skin and esophageal temperature in people aged >60 years while resting in hot and humid conditions (42°C, 30–70% RH; Gagnon et al., 2018). Future work should establish if this finding is persistent across other methods of increasing NO availability, and during exercise in the heat.

It has been established that increased aerobic fitness improves thermoregulation in middle aged adults (Stapleton et al., 2015a), and a low level of aerobic fitness has been identified as a risk factor for exertional heat illness in those aged <45 years (Lisman et al., 2014). Limited studies have investigated the effects of aerobic fitness on thermoregulation in individuals aged >65 years. Maintaining a high level of aerobic fitness throughout life protects total blood volume (Jones et al., 1997), and thus helps to maintain SkBF via increasing stroke volume resulting in increased cardiac output (Ho et al., 1997; Tankersley et al., 1991). High aerobic fitness is also associated with increased endothelial function via increased NO-dependent vasodilation in thermoneutral conditions (Taddei et al., 2000). Aerobic fitness training with carbohydrate and protein supplementation increased plasma volume in older (68 years) men, and reduced heart rate during exercise in the heat (Okazaki et al., 2009). Therefore, having a high aerobic fitness in old age should attenuate the decline in thermoregulation associated with aging. While these studies have investigated aerobic fitness and thermoregulatory capacity, the relationship between physical activity and thermoregulation in the elderly is not well understood.

### The Immune System

With aging there is an increase in the basal inflammatory state caused by prolonged exposure to antigen stress (Müller-Werdan, 2007) and an increasing amount of extra-nuclear DNA (Lan et al., 2019). Evidence suggests that with age there are alterations in the gut microbiota that lead to an increase in gut permeability and cytokine expression, and thus a reduced ability to repair damaged DNA (Guedj et al., 2020; Lam et al., 2012; Qi et al., 2017; Thevaranjan et al., 2017). The increase in permeability could, therefore, at least in part, relate to a decrease in gut microbiota diversity seen in aging (Biagi et al., 2010); however, there is debate about how aging affects microbiota diversity with some studies finding no decline in the elderly (Bian et al., 2017). Increased gut permeability can also occur during periods of passive and exercising heat stress (Selkirk et al., 2008; Snipe et al., 2018), which results in the release of endotoxin into the circulation (Selkirk et al., 2008). Endotoxin in the bloodstream triggers an immune response and inflammation (Aderem & Ulevitch, 2000). Given an increased level of inflammation and reduced immune functioning, the elderly are likely to have a reduced ability to cope with an increase in endotoxin released from the gut during heat stress. Although studies have examined the effect of heat stress on endotoxin, this work has mostly been conducted in animals (Hall et al., 2001) and young healthy participants (Selkirk et al., 2008). More cross-sectional and longitudinal research on the effects of heat stress on the immune system and circulating endotoxins within the elderly population is needed.

There appears to be an interaction between the immune system, gut permeability, and cardiovascular function. Patients with chronic heart failure have pathogenic gut flora overgrowth, increased gut permeability, and increased C-reactive protein (CRP) levels; this relationship correlated positively (intestinal permeability and right arterial pressure *r* = .55, *p* < .0001; CRP and intestinal permeability *r* = .78, *p* <. 0001; CRP and right arterial pressure *r* = .78, *p* < .0001) with severity of heart failure (Pasini et al., 2016). CRP is a strong predictor of cardiovascular disease and mortality (Proctor et al., 2015), and reduces NO availability (Shrivastava et al., 2015). Researchers should evaluate interventions for reducing inflammation or gut permeability to improve the immune response and reduce cardiovascular strain. Probiotics, prebiotics, and phenols can increase the diversity of gut microbiota and reduce gut permeability (Boulangé et al., 2016; Marchesi et al., 2016). Therefore probiotics, prebiotics, and phenols offer a potential dietary intervention for reducing aging-related inflammation and increasing gut integrity, and thus reducing cardiovascular strain.

### Behavioral Thermoregulation

Adapting behavior to the surrounding environment is an important aspect of thermoregulation (Flouris & Schlader, 2015). Behavior changes such as seeking shade or air-conditioned buildings, increasing fluid intake, removing clothing layers, or taking a cooling shower help to prevent the onset of heat illness (Harduar Morano et al., 2016). Waldock et al. (2018) explored the perceptual responses of the elderly during exercise in a hot environment and reported that despite increases in ambient temperature from 25°C to 35°C, and concomitant skin and core temperature increases, elderly participants did not perceive thermal comfort to be more uncomfortable when cycling at 6 METs for 30 min. Behavioral changes to environmental temperatures appear to be driven by thermal discomfort (Gagge et al., 1968). If the elderly have a diminished perception of discomfort despite being physiologically challenged, they may be reluctant to adapt their behavior(s) to reduce the thermal challenge. Studies are required to examine the role of perception of heat and physiological strain in the elderly to determine causal factors. As behavioral thermoregulation can prevent the onset of heat illness, improving the perceptual abilities of the elderly is important.

Altering workload, or pacing, during exercise and physical activity is a behavioral strategy that alters the thermal strain of an environment. Pacing research has shown that self-paced cycling output and running speed are reduced during exercise in hot compared with cool conditions, and this occurs prior to an increase in core temperature (Junge et al., 2016; Tucker & Noakes, 2009). When asked to cycle at a fixed rating of perceived exertion, power output dropped faster in 35°C than 25°C or 15°C (Tucker et al., 2006). Young men working in construction also self-pace during periods of extreme heat, maintaining an average heart rate below 110 bpm and a euthermic core temperature (Miller et al., 2011). This finding was consistent across workers with prior education on heat stress and those without (Miller et al., 2011). This outcome implies that a central control mechanism regulates exercise to protect against hyperthermia. Self-pacing strategies in temperate and hot conditions can be altered by manipulating neurotransmitters (Roelands et al., 2013). Self-paced workload increases with increases in dopamine resulting in a raised core temperature; alternatively, increased levels of serotonin and noradrenaline reduce workload and core temperature (Roelands et al., 2013; Roelands & Meeusen, 2010). Neurotransmitters clearly play a role in pacing behavior. Serotonin, noradrenaline, and dopamine levels decline with age (Peters, 2006; Rehman, 2001). Reduced levels of neurotransmitters will impact upon pacing behavior in an elderly population. Research is needed to establish how neurotransmitters impact pacing and thermal perception in the elderly during exercise in the heat.

Passive hyperthermia (core temperature 39.1°C) causes electroencephalographic (EEG) alterations in men (aged 35 years), theta wave power increases, causing an increased cognitive load, and an impaired ability to complete complicated cognitive tasks (Gaoua et al., 2018). EEG alterations are also seen during self-paced cycling in the heat (35°C, 60% RH) with reductions in alpha and beta activity in young (34 years) men (Périard et al., 2018). High alpha activity is associated with focus and ignoring irrelevant stimuli, while beta activity is required for mental readiness (Périard et al., 2018), thus heat stress during exercise hinders cognitive attention and readiness. In young men exercising in the heat, brain activity is closely correlated with core temperature and RPE; as the alpha/beta index increases, esophageal temperature and RPE increase (Nielsen et al., 2001; Nybo & Nielsen, 2001). There is no alteration in electromyographic activity, thus RPE is related to central fatigue rather than peripheral (Nybo & Nielsen, 2001). As RPE is involved with self-paced exercise intensity in the heat (Flouris & Schlader, 2015), it is important to understand how the age-related changes in EEG impacts this relationship. With aging there is a decline in overall EEG power, particularly in the alpha wave range (Vysata et al., 2012). A high level of aerobic fitness protects against declines in cognitive functioning (Barnes et al., 2003). Work is required to determine if the elderly’s attenuated ability to perceive exercise exertion is the result of a reduction in cognitive functioning.

## Heat Acclimation

Heat exposure during heat acclimation (HA) increases sweat rate, plasma volume, and thermal comfort, and reduces heart rate, core temperature, and skin temperature (Périard et al., 2015). These adaptations improve thermoregulatory capacity and are used by athletes before competition in hot environments. Six to eight days of HA is effective in highly trained and untrained older (≥ 50 years) populations (Best et al., 2014; Inoue et al., 1999). Benefits of HA can be seen from 5 days of exposure, although full adaptations typically require ~14 days (Pandolf, 1998). However, Daanen and Herweijer (2015) found that three days of acclimation training in women aged >75 years had no acclimation effect. This could be due to the limited number, and brevity (60 min/day) of exposures used. Daanen and Herweijer (2015) commented that the elderly participants struggled to complete the HA sessions despite not always increasing core temperature above 38.0°C, suggesting that the elderly would struggle to adhere to HA training.

**Figure 2.**
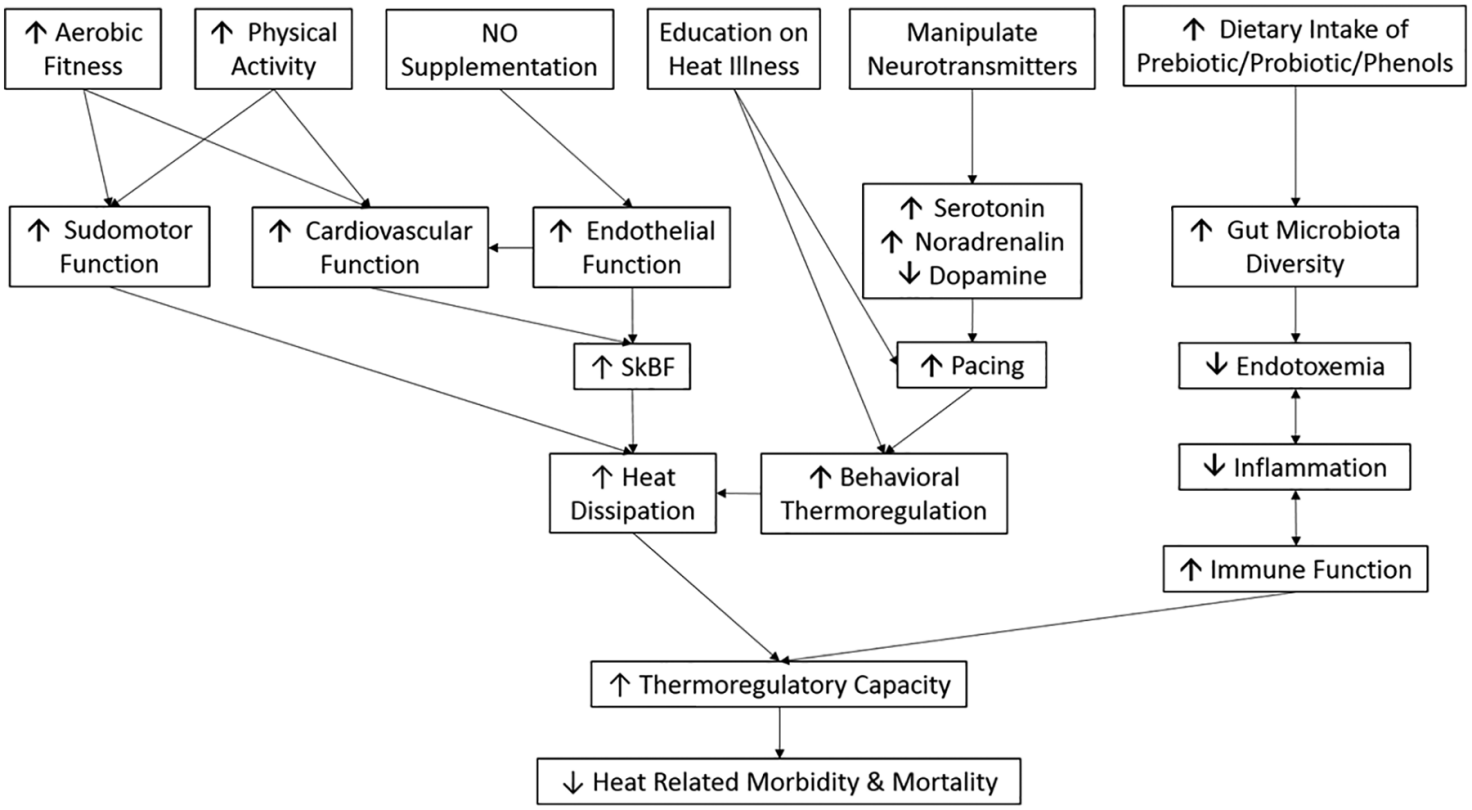
Proposed interventional strategies and mechanisms to improve thermoregulation in the elderly.

HA requires access to an environmental/heat chamber with core temperature recording equipment for safety. Prior to heat wave events HA could potentially improve thermoregulatory capacity of the elderly, thus reducing heat-related illness and death. However, access to HA facilities is expensive and impractical for the general public, limiting its use as a public health intervention. Encouraging HA strategies away from qualified supervision would be dangerous as excessive exposure will increase the risk of heat illness. Therefore, HA is not a practical solution to reducing the number of heat-related deaths in the elderly. Figure 2 provides an overview of possible effective and practical intervention strategies.

## Practical and Environmental Strategies

Several practical and economical strategies for reducing heat stress have been investigated. The use of electric fans is currently discouraged above ambient temperatures of 35°C by the World Health Organization (World Meteorological Organization & World Health Organization, 2015). However, there is evidence that electric fans are effective up to 42°C in young adults (Ravanelli et al., 2015). For older adults, fans usage is detrimental at such high temperatures (Gagnon et al., 2017); however, modeling predicts they are useful above 35°C, and would reduce or delay physiological strain for adults in severe heat waves (Jay et al., 2015). Research is needed in elderly human participants to confirm this modeling and investigate how the addition of other interventions affects fan usage limits.

Wearing a wet T-shirt during 2 hr of rested heat (42°C, 34% RH) exposure reduces the rise in core temperature in elderly (68 years) adults (Cramer et al., 2020). The addition of a fan eliminated the benefit of the wet T-shirt (Cramer et al., 2020), thus it is important to research how using multiple interventions interact with each other and not to assume an additive effect of multiple interventions. Wearing a wet T-shirt during a heat wave provides an inexpensive strategy that the public can implement with little effort required.

In sports it is well established that pre-cooling and per-cooling are effective in reducing physiological strain during exercise in the heat. Recent work demonstrates the benefit of combining cooling techniques during heat exposure (35°C) for young (22 years) men (Nakamura et al., 2020). After reaching a rectal temperature of 38.5°C, forearm immersion in cold water (10°C) combined with ice slurry (-1°C) ingestion cooled rectal temperature more quickly than ice slurry ingestion alone, after 15 minutes of cooling rectal temperature was 37.9°C for combined cooling and 38.3°C for ice slurry ingestion alone (Nakamura et al., 2020). The combined use of external and internal cooling is of added benefit over internal or external alone. While much research has been conducted on young adults in a sporting situation, there is a dearth of research investigating the effects of cold-water immersion and ice slurry ingestion on the elderly.

Alongside personal interventions, environmental and structural alterations can also reduce heat exposure. The urban heat island effect is a common phenomenon in urbanized areas, and maintaining spaces with natural vegetation rather than paving will reduce heat radiation from these surface and lower ambient temperatures (Dwivedi & Mohan, 2018). Building design can also be adapted to mitigate the impact of heat waves: the use of shutters, triple glazing, and reflective roofs are effective methods of reducing overheating in care facilities (Gupta & Gregg, 2017). Town and buildings planners, as well as individuals, should look to implement these strategies and consider the long-term effects of global warming on their designs and homes.

## Conclusion

With an aging population and increases in the severity and frequency of extreme weather events, heat illness will become a major public health issue with a global impact. A key issue is the apparent lack of perception of thermal and physical strain during heat exposure in elderly individuals, and its influence over behavioral thermoregulation. Adapting behavior during heat exposure has the potential to eliminate the risk of heat illness, thus research is vital in establishing how the elderly behave during heat exposure. The influence of neurotransmitters and cognitive functioning on the perception of thermal and physical strain also require further examination. Reductions in cardiovascular and sudomotor function are well understood; however, the potential for NO supplementation to improve this functioning in an elderly population warrants additional investigation. While gut-related endotoxemia is a mechanism implicated in the induction of heat stroke, additional research is needed to examine how this mechanism affects the elderly and its potential role in cardiovascular strain.
